# Poly-D,L-Lactic Acid Fillers Increase Subcutaneous Adipose Tissue Volume by Promoting Adipogenesis in Aged Animal Skin

**DOI:** 10.3390/ijms252312739

**Published:** 2024-11-27

**Authors:** Kyung-A Byun, Suk Bae Seo, Seyeon Oh, Jong-Won Jang, Kuk Hui Son, Kyunghee Byun

**Affiliations:** 1 Department of Anatomy & Cell Biology, College of Medicine, Gachon University, Incheon 21936, Republic of Korea; 2 LIBON Inc., Incheon 22006, Republic of Korea; 3 Functional Cellular Networks Laboratory, Lee Gil Ya Cancer and Diabetes Institute, Gachon University, Incheon 21999, Republic of Korea; 4 SeoAh Song Dermatologic Clinic, Seoul 05557, Republic of Korea; 5 Department of Health Sciences and Technology, Gachon Advanced Institute for Health & Sciences and Technology (GAIHST), Gachon University, Incheon 21999, Republic of Korea; 6 Department of Thoracic and Cardiovascular Surgery, Gachon University Gil Medical Center, Gachon University, Incheon 21565, Republic of Korea

**Keywords:** adipogenesis, adipose-derived stem cells, poly-D,L-lactic acid, subcutaneous white adipose tissue

## Abstract

During aging, subcutaneous white adipose tissue (sWAT) thickness and the adipogenic potential of adipose-derived stem cells (ASCs) decline. Poly-D,L-lactic acid (PDLLA) fillers are commonly used to restore diminished facial volume. Piezo1 increases polarizing macrophages towards the M2 phenotype, which promotes the secretion of fibroblast growth factor 2 (FGF2), thereby increasing ASC survival. This study evaluated whether PDLLA enhances adipogenesis in ASCs by modulating M2 polarization in an in vitro senescence model and in aged animals. Lipopolysaccharide (LPS)-induced senescent macrophages showed decreased Piezo1, which was upregulated by PDLLA. CD163 (an M2 marker) and FGF2 were downregulated in senescent macrophages but were upregulated by PDLLA. We evaluated whether reduced FGF2 secretion from senescent macrophages affects ASCs by applying conditioned media (CM) from macrophage cultures to ASCs. CM from senescent macrophages decreased ERK1/2 and proliferation in ASCs, both of which were restored by CM from PDLLA-stimulated senescent macrophages. Adipogenesis inducers (PPAR-γ and C/EBP-α) were downregulated by CM from senescent macrophages but upregulated by CM from PDLLA-stimulated senescent macrophages in ASCs. Similar patterns were observed in aged animal adipose tissue. PDLLA increased Piezo1 activity, M2 polarization, and FGF2 levels. PDLLA also enhanced ERK1/2, cell proliferation, PPAR-γ, and C/EBP-α expression, leading to increased adipose tissue thickness. In conclusion, our study showed that PDLLA increased adipose tissue thickness by modulating adipogenesis.

## 1. Introduction

Macrophages play an essential role in various tissue repair processes [1]. In response to microenvironmental signals, macrophages undergo polarization into two main types: M1 and M2 [2,3,4]. M1 macrophages primarily secrete pro-inflammatory cytokines, whereas M2 macrophages release anti-inflammatory cytokines [4,5]. M2 macrophages promote fibroblast proliferation and facilitate tissue remodeling by enhancing extracellular matrix (ECM) synthesis [6]. They also secrete various stimulatory factors, such as transforming growth factor-beta (TGF-β), fibroblast growth factor 2 (FGF2) [7], platelet-derived growth factor [8], and galectin-3 [9], which promote fibroblast proliferation, survival, and differentiation into myofibroblasts [10]. These factors collectively lead to increased ECM formation [10].

Macrophage polarization is influenced by both photoaging and chronological aging. Photoaging alters the balance between M1 and M2 macrophages, contributing to chronic inflammation [11]. Ultraviolet radiation drives macrophages towards an M1 phenotype, inducing inflammation and fibroblast senescence [12]. During aging, senescent fibroblast accumulation increases in the dermis [13]. Senescent fibroblasts exhibit reduced collagen synthesis and increased expression levels of matrix metalloproteinase (MMP) 1, MMP3, and MMP9, which degrade collagen fibers [14].

Macrophages also play a role in collagen synthesis, which can be enhanced by various dermal fillers. Dermal fillers induce a subclinical foreign body reaction in which immune cells, including macrophages, stimulate fibroblast recruitment and collagen synthesis by increasing TGF-β levels [15,16,17]. Our previous work demonstrated that pIoly-L-lactic acid (PLLA) fillers increase macrophage polarization towards the M2 phenotype, which is associated with elevated interleukin-10 (IL-10) and TGF-β levels [18]. These cytokines enhance fibroblast proliferation and collagen synthesis in senescent fibroblasts and aged skin [18]. Our group also demonstrated that poly-D,L-lactic acid (PDLLA) increases nuclear factor erythroid 2-related factor 2 (NRF2) expression, which enhances M2 polarization and IL-10 secretion in senescent macrophages [19]. Elevated IL-10 levels reduce senescence in adipose-derived stem cells (ASCs), subsequently promoting the secretion of TGF-β and FGF2 [19]. These cytokines are linked to increased collagen synthesis in senescent fibroblasts [19]. Various stimuli, including IL-4, IL-13, IL-10, and TGF-β, promote M2 macrophage polarization [20].

The activation of ion channels such as Piezo-type mechanosensitive ion channel component 1 (Piezo1) also favors M2 polarization [21]. Piezo1 channels respond to mechanical stress, such as changes in substrate stiffness, static pressure, membrane stretching, and shear flow, facilitating extracellular Ca^2+^ influx into the cytosol [22,23,24]. Mechanical stretching at 10% and 0.5 Hz promotes M2 polarization, increasing TGF-β1 secretion and enhancing the proliferation and migration of bone marrow mesenchymal stem cells [21].

Facial subcutaneous white adipose tissue (sWAT) undergoes changes during aging; thus, sWAT modulation is a target for skin rejuvenation [25,26,27,28]. Aging reduces sWAT volume and alters its connections with collagen networks, which leads to skin wrinkling [29,30]. The proliferation and differentiation capacities of preadipocytes, as well as their number, decrease during aging [27,31,32,33]. The expression of peroxisome proliferator-activated receptor-γ (PPAR-γ), a key factor in adipogenesis, also declines during aging [31]. These changes contribute to structural instability, which is associated with wrinkle formation [34]. 

Recent studies suggest that dermal fillers can rejuvenate skin by promoting ASC proliferation and mature adipocyte expansion [35]. Hyaluronic acid has been shown to increase ASC proliferation, supporting adipose tissue formation [36]. There is also speculation that the loss of volume after filler absorption could be mitigated by promoting adipose tissue formation [36].

Adipogenesis, the differentiation of preadipocytes into mature adipocytes, is regulated by key adipogenic genes such as *PPAR-γ*, CCAAT/enhancer-binding protein-α (*C/EBP-α*), and sterol regulatory element-binding protein-1c (*SREBP-1c*) [37]. FGF2 stimulates ASCs, enhancing their proliferation, self-renewal, and differentiation capacities [38,39]. When administered to adipocyte precursors, FGF2 increases PPAR-γ expression, thereby promoting adipocyte differentiation [38]. FGF2 also enhances the proliferation of adipocyte precursors [40] and activates extracellular signal-regulated kinases (ERK) 1/2, which subsequently increase adipogenesis in its early stages [41]. Although our group previously demonstrated that PDLLA increases collagen synthesis by modulating M2 polarization in aged skin [19], it remains unclear whether PDLLA can also enhance adipogenesis, potentially prolonging the volume augmentation effects of fillers.

This study evaluated whether PDLLA promotes adipogenesis in ASCs through M2 polarization modulation in a senescent in vitro model and in aged animals. We hypothesized that PDLLA induces mechanical stimuli, which activate Piezo1 and M2 polarization. Increased M2 polarization leads to FGF2 secretion, which subsequently elevates ERK1/2 levels. Elevated ERK1/2 results in ASC proliferation and differentiation into mature adipocytes in aged skin. These mature adipocytes contribute to skin rejuvenation by increasing sWAT thickness.

## 2. Results

### 2.1. PDLLA Increases Piezo1 Expression and M2 Polarization in Senescent Macrophages

**Figure 1:**
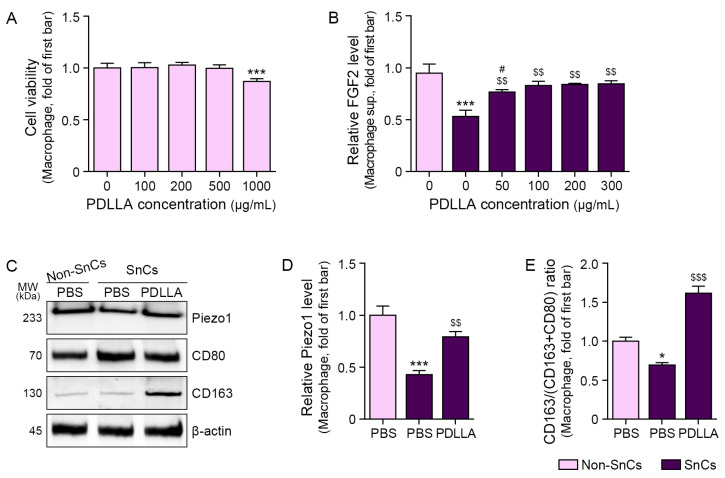
Regulation of Piezo1 expression and M2 polarization through PDLLA treatment in macrophages. (**A**) Cell viability in macrophages treated with various concentrations of PDLLA. (**B**) FGF2 secretion in the supernatant of senescent macrophages treated with various concentrations of PDLLA. (**C**–**E**) Piezo1 expression and CD163 to (CD80 + CD163) ratio in senescent macrophages treated with PDLLA. Data are presented as the mean ± standard deviation of three independent experiments. *, *p* < 0.05 and ***, *p* < 0.001 vs. first bar; $$, *p* < 0.01 and $$$, *p* < 0.001 vs. second bar; #, *p* < 0.05 vs. fourth bar (Mann–Whitney U test). FGF2, fibroblast growth factor 2; PBS, phosphate-buffered saline; PDLLA, poly-D,L-lactic acid; sup, supernatant.

The optimal concentration of PDLLA for in vitro experiments was determined based on its effects on cell viability and FGF2 expression. PDLLA at 100, 200, and 500 μg/mL did not impact macrophage viability relative to cells treated with phosphate-buffered saline (PBS; pH7.2, sodium counter ion). However, macrophage viability declined at concentrations above 1000 μg/mL (Figure 1A). Macrophage senescence was induced in THP-1 cells by lipopolysaccharide (LPS) treatment, as established in previous studies [42,43,44,45,46]. Senescence markers p21 and p16 were elevated after LPS treatment (Figure S1). LPS-treated macrophages showed reduced FGF2 secretion, whereas PDLLA treatment increased FGF2 levels. There was no significant difference in the enhancement of FGF2 among PDLLA concentrations of 100, 200, and 300 μg/mL. Therefore, 100 μg/mL PDLLA was selected for subsequent experiments (Figure 1B). Piezo1 expression was downregulated in senescent macrophages but upregulated after PDLLA treatment (Figure 1C,D).

Macrophage surface markers such as CD80 are frequently used as M1 markers; however, CD163 or CD80 are used as M2 markers [6,47,48]. The ratio of CD80/CD163 or CD80/CD206 has been used to assess macrophage polarization [49,50].

In this study, M2 polarization was assessed using the ratio of CD163 to the total CD163 and CD80. The CD163/(CD163 + CD80) ratio declined in senescent macrophages and was restored by PDLLA treatment (Figure 1C,E).

### 2.2. PDLLA Increases ASC Proliferation and Differentiation into Mature Adipocytes

To assess whether PDLLA modulates ASCs via macrophages, we developed an in vitro model in which conditioned media (CM) from senescent macrophages were applied to ASCs. CM from non-senescent macrophages is referred to as CM_Non-Sncs_, CM from PBS-treated senescent macrophages as CM_Sncs_, and CM from PDLLA-stimulated senescent macrophages as CM_Sncs/PDLLA_ (Figure S2).

**Figure 2:**
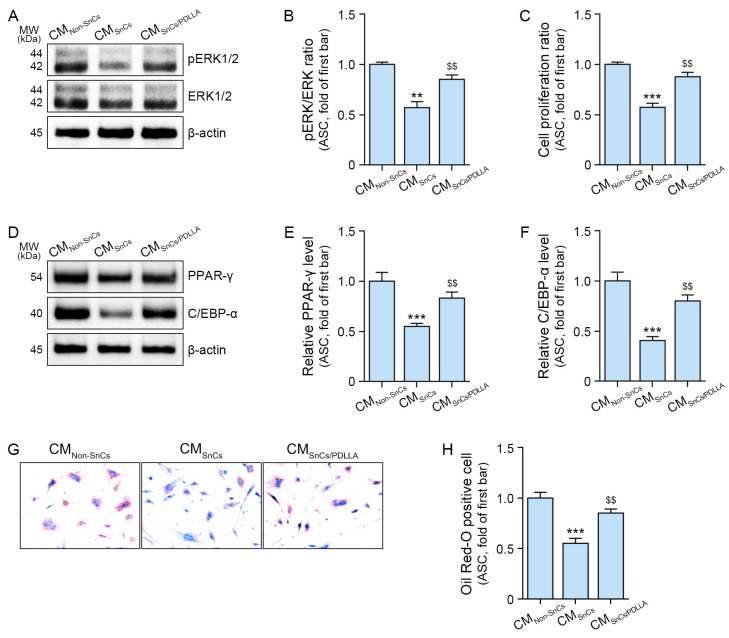
Regulation of ASC proliferation and differentiation into mature adipocytes by PDLLA treatment. (**A,B**) Expression ratio of pERK1/2 to ERK1/2 in ASCs treated with CM_Non-Sncs_, CM_Sncs_, or CM_Sncs/PDLLA_. (**C**) Cell proliferation of ASCs treated with CM_Non-Sncs_, CM_Sncs_, or CM_Sncs/PDLLA_. (**D**–**F**) Expression of PPAR-γ and C/EBP-α in mature adipocytes differentiated from ASCs treated with CM_Non-Sncs_, CM_Sncs_, or CM_Sncs/PDLLA_. (**G**,**H**) Oil Red O staining in mature adipocytes differentiated from ASCs treated with CM_Non-Sncs,_ CM_Sncs_, or CM_Sncs/PDLLA_. Data are presented as the mean ± standard deviation of three independent experiments. **, *p* < 0.01 and ***, *p* < 0.001 vs. first bar; $$, *p* < 0.01 vs. second bar; vs. fourth bar (Mann–Whitney U test). C/EBP-α, CCAAT/enhancer-binding protein-α; CM_Non-Sncs_, CM from PBS-treated non-senescent macrophages; CM_Sncs_, CM from PBS-treated senescent macrophages; CM_Sncs/PDLLA_, CM from PDLLA-treated senescent macrophages; ERK, extracellular signal-regulated kinase; PBS, phosphate-buffered saline; PDLLA, poly-D,L-lactic acid; PPAR-γ, proliferator-activated receptor-γ.

The pERK1/2 to ERK1/2 expression ratio decreased in ASCs treated with CM_Sncs_ but increased when treated with CM_Sncs/PDLLA_ (Figure 2A,B). ASC proliferation, assessed using the cell counting kit-8 (CCK-8) assay, decreased with CM_Sncs_ treatment but increased with CM_Sncs/PDLLA_ (Figure 2C).

The adipogenesis factors PPAR-γ and C/EBP-α were reduced by CM_Sncs_ but increased by CM_Sncs/PDLLA_ (Figure 2D–F). Differentiation into mature adipocytes was assessed using Oil Red O staining [51]. The number of Oil Red O-positive cells decreased with CM_Sncs_ treatment but increased with CM_Sncs/PDLLA_ (Figure 2G,H). These results suggest that PDLLA modulates macrophages, which then influence ASCs, causing increased ASC proliferation and differentiation into mature adipocytes.

### 2.3. PDLLA Increases Expression of Piezo1, M2 Polarization Markers, FGF2, and Adipogenesis Factors in sWAT of Aged Animals

**Figure 3:**
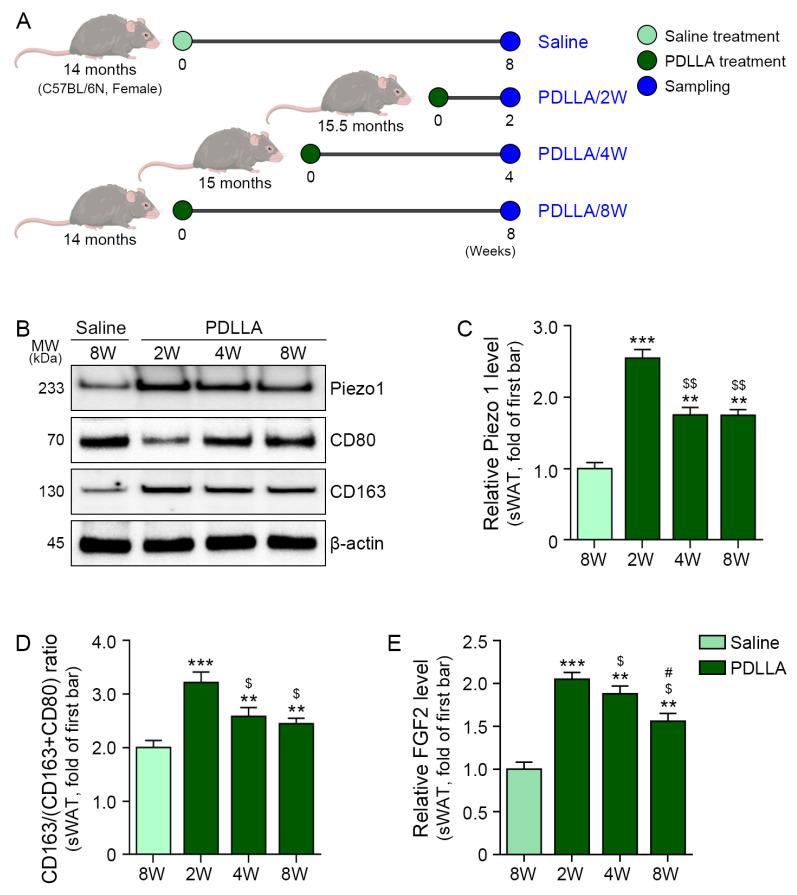
Regulation of Piezo1 expression, M2 polarization, and FGF2 expression by PDLLA treatment in sWAT of aged animals. (**A**) Schematic diagram of PDLLA treatment in aged mice. (**B**–**D**) Piezo1 expression and CD163 to (CD80 + CD163) ratio in sWAT of aged mice treated with PDLLA. (**E**) FGF2 expression in sWAT of aged mice treated with PDLLA. Data are presented as the mean ± standard deviation of three independent experiments. **, *p* < 0.01 and ***, *p* < 0.001 vs. first bar; $, *p* < 0.05 and $$, *p* < 0.01 vs. second bar; #, *p* < 0.05 vs. third bar (Mann–Whitney U test). FGF2, fibroblast growth factor 2; PBS, phosphate-buffered saline; PDLLA, poly-D,L-lactic acid; W, weeks.

In the in vivo mouse model (Figure 3A), Piezo1 expression increased in the sWAT after PDLLA injection, peaking at 2 weeks post-injection. The CD163/(CD80 + CD163) ratio also increased in aged sWAT after PDLLA injection, peaking at 2 weeks post-injection (Figure 3B–D). FGF2 levels increased in aged sWAT after PDLLA injection, peaking at 2 weeks post-injection (Figure 3E).

**Figure 4:**
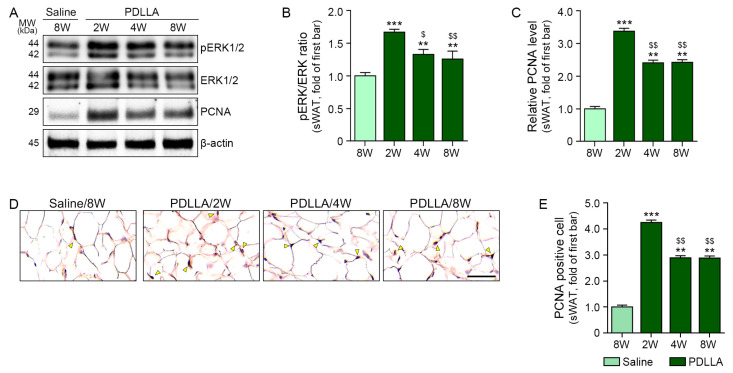
Regulation of proliferation marker by PDLLA treatment in sWAT of aged animals. (**A**–**C**) pERK1/2 to ERK1/2 ratio and PCNA expression in sWAT of aged mice treated with PDLLA. (**D**,**E**) PCNA expression in sWAT of aged mice treated with PDLLA. Yellow mark is a positive signal. Data are presented as the mean ± standard deviation of three independent experiments. **, *p* < 0.01 and ***, *p* < 0.001 vs. first bar; $, *p* < 0.05 and $$, *p* < 0.01 vs. second bar (Mann–Whitney U test). ERK, extracellular signal-regulated kinase; PBS, phosphate-buffered saline; PCNA, proliferating cell nuclear antigen; PDLLA, poly-D,L-lactic acid.

The pERK1/2 to ERK1/2 ratio also rose after PDLLA injection in sWAT, such that the highest levels were observed at 2 weeks. Cell proliferation was assessed using Western blotting of PCNA, which showed an increase in PCNA expression in sWAT after PDLLA injection; similarly, the peak occurred at 2 weeks (Figure 4A–C).

PCNA staining of whole skin, including sWAT, was performed to determine whether cell proliferation was more pronounced in sWAT than in other skin layers, such as the epidermis, after PDLLA treatment (Figure S3 and Figure 4D,E). PCNA intensity in sWAT increased after PDLLA injection in aged skin; the highest levels were evident at 2 weeks post-injection (Figure 4D,E).

**Figure 5:**
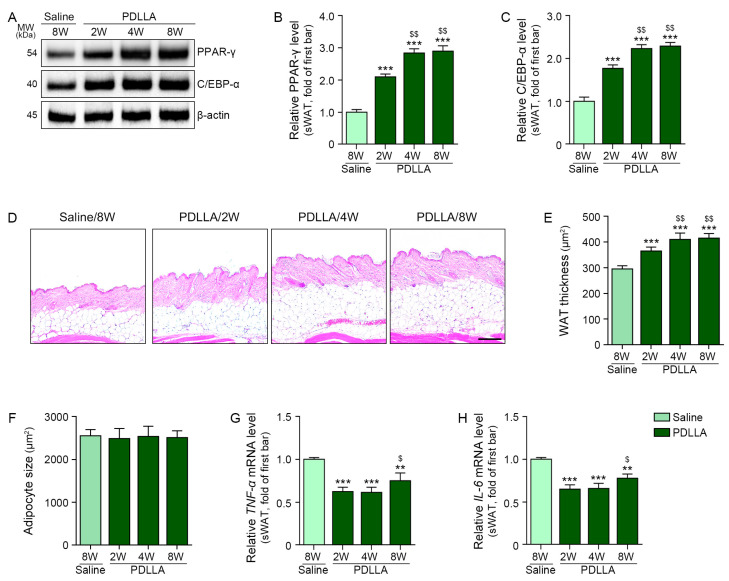
Regulation of adipogenesis factors by PDLLA treatment in sWAT of aged animals. (**A**–**C**) Expression levels of PPAR-γ and C/EBP-α in sWAT of aged mice treated with PDLLA. (**D**) Hematoxylin and eosin staining of sWAT in aged mice treated with PDLLA. (**E**,**F**) Adipose tissue thickness and adipocyte size in sWAT of aged mice treated with PDLLA. (**G,H**) *TNF-α* and *IL-6* mRNA level in sWAT of aged mice treated with PDLLA. Data are presented as the mean ± standard deviation of three independent experiments. **, *p* < 0.01 and ***, *p* < 0.001 vs. first bar; $, *p* < 0.05 and $$, *p* < 0.01 vs. second bar (Mann–Whitney U test). C/EBP-α, CCAAT/enhancer-binding protein-α; IL-6, interleukin-6; PBS, phosphate-buffered saline; PDLLA, poly-D,L-lactic acid; PPAR-γ, proliferator-activated receptor-γ; TNF-α, tumor necrosis factor-α.

PPAR-γ and C/EBP-α expression levels increased in sWAT after PDLLA injection; both levels rose over time (Figure 5A–C). sWAT thickness also increased with PDLLA injection, revealing a time-dependent effect (Figure 5D,E). However, adipocyte size remained unchanged after PDLLA treatment (Figure 5D,F). The levels of pro-inflammatory cytokines such as tumor necrosis factor-α (TNF-α) and interleukin (IL)-6 decreased after PDLLA treatment. At 8 weeks after PDLLA injection, both the levels of TNF-α and IL-6 were higher than those 2 or 4 weeks after PDLLA injection (Figure 5G,H).

## 3. Discussion

Aging-induced skin atrophy is associated with the loss of ECM components, epidermal thinning, and dermal elastosis [52]. Facial adipose tissue also undergoes aging-related changes, resulting in volume loss and alterations in facial shape [52]. Decreased midface volume is a characteristic feature of the aged face [53]. To restore youthful facial contours, adipose tissue grafting has been attempted [52]. Although particle fat transplantation offers rapid and convenient volume restoration [54], this procedure can result in complications, such as irregular fat accumulation, facial swelling, fat cysts, and lipogranulomas due to excessive release of lipid droplets [55,56]. Additionally, because aging affects adipose tissue itself, fat transplantation in aged skin may have limitations, including a reduced effect on skin rejuvenation.

Adipose tissue comprises two main components: the adipocyte fraction and the stromovascular fraction (SVF). The adipocyte fraction primarily consists of mature adipocytes; the SVF contains various cell types, including fibroblasts, pericytes, macrophages, ASCs, and other stem cell phenotypes [57]. The function of adipose progenitors, or ASCs, is essential for adipose tissue renewal, expansion, and plasticity [58]. However, aging decreases the proliferative and differentiation abilities of adipose progenitors [59,60]. Declines in ASC proliferation begin around age 30 and become more pronounced by age 50 [61]. ASCs can be obtained through minimally invasive procedures; thus, ASC injections have been explored for tissue regeneration, including skin rejuvenation [21,62]. However, ASC injections have limitations, such as low engraftment rates and reduced biological activity post-injection [63,64]. Moreover, ASC biological activity varies according to donor age and health status [65,66]. An effective approach for regenerating skin adipose tissue to promote skin rejuvenation is needed to overcome the limitations of ASC or SVF injections.

Piezo1 is activated by changes in ECM stiffness and contributes to fibrosis by increasing ECM production [67,68]. Piezo1 activation increases intracellular Ca^2+^ levels, triggering signaling pathways such as protein kinase B (AKT)/mechanistic target of rapamycin (mTOR) and p38 mitogen-activated protein kinase, which are associated with cell proliferation [21,69,70,71,72].

Our group previously demonstrated that PLLA promotes fibroblast proliferation through AKT/mTOR pathway activation, ultimately enhancing collagen synthesis [73]. The increase in AKT/mTOR was linked to Piezo1 activation, given that the Piezo1 inhibitor GsMTx4 reduced PLLA-induced AKT/mTOR activity [73]. This finding suggests that PLLA activates Piezo1 to stimulate tissue collagen synthesis [73]. However, the precise mechanism by which PLLA enhances Piezo1 activity remains unclear. We speculate that PLLA-injection-induced changes to ECM stiffness result in Piezo1 activation [73]. 

The activation of Piezo1 also plays a role in M2 polarization, which increases TGF-β levels and promotes stem cell proliferation and bone formation [21]. M2 macrophages secrete both FGF2 and TGF-β, which together support ECM formation [7,10]. This study evaluated whether PDLLA filler increases Piezo1 activity and M2 polarization to enhance FGF2 levels. In senescent macrophages, Piezo1 activity and M2 polarization were reduced; however, these parameters increased after PDLLA treatment. FGF2 secretion from senescent macrophages also initially decreased; it increased after PDLLA treatment. FGF2 is an essential factor for ASC proliferation and adipogenesis [38]. When administered to human ASCs, FGF2 activates ERK, JNK, and p38 pathways, enhancing cell proliferation [74]. Senescent ASCs exhibit increased doubling times and decreased FGF2 secretion, which reduces the autocrine effects of FGF2 and, consequently, cell proliferation [39].

To determine whether reduced FGF2 secretion from senescent macrophages affects ASCs, we conducted an in vitro experiment. When ASCs were treated with CM from senescent macrophages, ERK1/2 activity and proliferation ratios decreased. However, both ERK1/2 activity and proliferation were restored when ASCs were treated with CM from PDLLA-stimulated senescent macrophages. Adipogenesis inducers such as PPAR-γ and C/EBP-α, which were reduced by CM from senescent macrophages, increased when CM from PDLLA-stimulated senescent macrophages was applied to ASCs. Similarly, the number of mature adipocytes decreased when ASCs were treated with CM from senescent macrophages but increased with CM from PDLLA-stimulated macrophages. These results suggest that PDLLA enhances Piezo1 activity and M2 polarization, leading to increased FGF2 secretion from senescent macrophages. The elevated FGF2 levels promote ASC proliferation and adipogenesis. Similar patterns were observed in the animal model. In aged animal adipose tissue, PDLLA treatment increased Piezo1 activity, M2 polarization, and FGF2 levels. ERK1/2 activity and cell proliferation also increased with PDLLA, along with elevated expression of PPAR-γ and C/EBP-α. Additionally, adipose tissue thickness increased with PDLLA treatment, but adipocyte size remained unchanged.

Adipose tissue expansion can occur through either increased adipocyte size (hypertrophic expansion) or an increased number of adipocytes (hyperplastic expansion) [75,76,77]. Whereas hyperplastic expansion is generally considered a healthy form of adipose tissue growth, hypertrophic expansion can lead to tissue dysfunction and is associated with chronic inflammation [75,76,77]. Soft tissue fillers are reported to increase tissue volume by promoting ASC adipogenesis and mature adipocyte hypertrophy [1]. Considering the 4–5-day doubling time of ASCs in vitro, volume increases through adipogenesis could occur within a short period after filler injection [35]. Our animal study demonstrated that M2 polarization and FGF2 expression in sWAT were highest at 2 weeks post-PDLLA-injection. ERK1/2 activity and the cell proliferation ratio were also highest at 2 weeks. However, adipogenic gene expression and sWAT thickness continued to increase over time and were highest at 8 weeks post-injection.

Because the restoration of youthful facial contours through volume restoration is essential for facial rejuvenation, effective methods to achieve this outcome are needed. PDLLA fillers are primarily known to restore facial volume by stimulating neo-collagen synthesis. However, our study demonstrated that PDLLA may also increase facial volume by enhancing sWAT thickness. PDLLA promoted ASC proliferation and adipogenesis in an in vitro senescence model. Moreover, animal studies showed that PDLLA increased sWAT thickness without affecting adipocyte size. PDLLA decreased TNF-α and IL-6 while increasing sWAT thickness. These results suggest that PDLLA increased sWAT with little inflammation.

The increase in adipogenesis may be mediated by macrophages, but the exact mechanism by which PDLLA induces M2 polarization was not fully clarified in this study. Because Piezo1 activation has been shown to drive M2 polarization and our findings indicate that PDLLA activates Piezo1 along with M2 polarization, it is plausible that PDLLA-induced Piezo1 activation contributes to increased M2 polarization. Future studies should investigate the precise mechanism by which PDLLA activates Piezo1 and its relationship to M2 polarization. The PDLLA filler we used in the study contained 0.6% hyaluronic acid solution at an 85:15 ratio. Thus, it is possible that hyaluronic acid also affected the Piezo1 activity. In this study, we did not compare the effect of PDLLA filler on Piezo1 with single hyaluronic acid treatment. The differences in effects between PDLLA and hyaluronic acid should be investigated in future research.

Although our study did not directly address these mechanisms, the findings suggest that PDLLA can serve as an alternative approach for increasing facial volume through adipogenesis during aging.

## 4. Materials and Methods

### 4.1. PDLLA Preparation

PDLLA (Juvelook volume; VAIM Co., Ltd., Daejeon, Republic of Korea) was dissolved in a 1:9 mixture of ethylene carbonate and dimethyl sulfoxide (Sigma-Aldrich, St. Louis, MO, USA). This solution was sprayed into cold n-hexane (below −10 °C) to form a solvent/polymer mixture, which was then combined with distilled water and filtered to remove the solvent, yielding solid PDLLA particles (~45 µm in size). The particles were dried and mixed with a 0.6% hyaluronic acid solution at an 85:15 ratio, aliquoted into vials, lyophilized, and sterilized with ethylene oxide gas.

### 4.2. In Vitro Experiments

#### 4.2.1. Cell Culture

THP-1 human monocyte cells, obtained from the Korea Cell Line Bank (Seoul, Republic of Korea), were cultured in RPMI-1640 medium (Welgene, Gyeongsan, Republic of Korea) supplemented with 10% fetal bovine serum (FBS; Gibco, Waltham, MA, USA) and 1% penicillin–streptomycin (Welgene). These cells were differentiated into macrophages through treatment with 100 ng/mL of 4β-phorbol-12-myristate-13-acetate (PMA; Sigma-Aldrich), and further experiments were initiated 24 h after differentiation [78].

Adipose-derived mesenchymal stem cells (AD-MSCs) were acquired from CEFO (Seoul, Republic of Korea) and were cultured in CEFOgro™ Human MSC growth medium, supplemented with CEFOgro™ human-adipose-tissue-derived MSC kit components.

All cell cultures were maintained at 37 °C in a humidified atmosphere containing 5% CO_2_. Cells were used for experimental purposes once they reached approximately 80% confluence.

#### 4.2.2. Experimental Design for PDLLA Treatment

To investigate the effects of PDLLA on macrophage senescence, THP-1-derived macrophages were treated with 1 μg/mL lipopolysaccharide (LPS) for 24 h to induce senescence. These senescent macrophages were subsequently exposed to PDLLA at concentrations ranging from 0 to 300 μg/mL for an additional 48 h. After testing various concentrations, 100 μg/mL was identified as the optimal concentration for further cell experiments. For the main experiments, proteins were extracted from cell lysates, and the culture medium was collected for AD-MSC culture. The culture medium containing conditioned media from the treated macrophages was centrifuged at 300× *g* for 5 min to remove cell debris (Figure S1A).

To assess the adipogenic potential of PDLLA, AD-MSCs were treated with a StemPro Adipocyte Differentiation Basal Medium (Gibco) that included StemPro Adipocyte Supplement (Gibco) and Gentamicin (Gibco) every 3 days over a 12-day period. During differentiation, the AD-MSCs were exposed to a mixture of conditioned media from macrophages (derived as above) and differentiation medium in a 1:1 ratio. This experiment was performed according to the StemPro™ Adipogenesis Differentiation Kit (Gibco) manufacturer’s protocol (Figure S2).

### 4.3. In Vivo Experiments

#### 4.3.1. Mouse Model and Maintenance

Six-week-old C57BL/6 mice were purchased from Orient Bio (Seongnam, Republic of Korea). The animals were housed in a controlled environment with a constant temperature of 20–24 °C and humidity levels of 45–55%. Mice were allowed free access to standard laboratory food and water throughout the study. At 14 months of age, the animals were randomly divided into four groups for experimental treatment. This study was conducted with approval from the Gachon University Animal Experiment Ethics Committee (IACUC approval number LCDI-2023-0154). 

#### 4.3.2. Experimental Design for PDLLA Injections

The stabilized mice were allocated into four experimental groups. The control group (group 1) received an injection of normal saline, while the other three groups (groups 2, 3, and 4) received a single PDLLA injection in a 2 cm × 2 cm area on the back using a 27 G needle. The injection volume for each PDLLA solution was 500 μL. In group 2, PDLLA was injected at 15.5 months of age, in group 3 at 15 months, and in group 4 at 14 months to synchronize sample collection across time points. Skin and sWAT samples were harvested at different time intervals: group 2 after 14 days, group 3 after 28 days, and groups 1 and 4 after 56 days post-injection (Figure 3A).

### 4.4. Sample Preparation

#### 4.4.1. RNA Extraction and cDNA Synthesis

RNA was extracted from tissue samples using the RNAiso reagent (TAKARA, Tokyo, Japan). The RNA concentration was quantified using a Nanodrop spectrophotometer (Thermo Fisher Scientific, Waltham, MA, USA), and 1 μg of total RNA was used to synthesize complementary DNA (cDNA) following the manufacturer’s protocol.

#### 4.4.2. Protein Isolation and Concentration Quantitation

Proteins were isolated using EzRIPA buffer (ATTO Corporation, Tokyo, Japan), and their concentrations were measured using a bicinchoninic acid (BCA) assay kit (Thermo Fisher Scientific).

#### 4.4.3. Paraffin-Embedded Skin Tissue Blocks

Tissue samples were fixed with 4% paraformaldehyde (Sigma-Aldrich) for 72 h, processed in a tissue processor (Leica, Wetzlar, Germany), and embedded in paraffin blocks after dehydration. The tissue was sectioned into 7 µm slices with a microtome and mounted onto slides by incubating at 60 °C overnight.

### 4.5. Cell Viability Assessment

To evaluate PDLLA cytotoxicity, monocytes were cultured in a 96-well plate, differentiated with PMA, and treated with increasing concentrations of PDLLA (100–2000 μg/mL) for 24 h. After treatment, a CCK-8 reagent (TransGen Biotech Co., Ltd., Beijing, China) was added to each well, and optical density was measured at 450 nm after 2 h of incubation. Each condition was tested in triplicate.

### 4.6. Quantitative Reverse Transcription Polymerase Chain Reaction (RT-qPCR)

RT-qPCR was performed using a QuantStudio™ 3 Real-Time PCR System (Thermo Fisher Scientific) with SYBR green premix (TAKARA). Reaction conditions included initial denaturation at 95 °C for 10 min, followed by 40 cycles at 95 °C for 15 s, 60 °C for 1 min, and another 15 s at 95 °C. Gene expression levels were calculated using the ΔΔCT method and normalized to ACTB. The forward and reverse primers are listed in Table S1.

### 4.7. Enzyme-Linked Immunosorbent Assay

Microplates were coated with a capture antibody in a carbonate–bicarbonate buffer (pH9.6, sodium counter ion), blocked with skim milk to prevent nonspecific binding, and incubated with the protein sample. After additional washes, primary antibodies (diluted in PBS; Table S2) were added and incubated overnight at 4 °C. After further washing, a horseradish-peroxidase-conjugated secondary antibody (1:10,000; Vector Laboratories, Newark, CA, USA) was added and incubated at room temperature for 3 h. To detect protein expression, tetramethylbenzidine solution (Sigma-Aldrich) was added and incubated for 10 min at room temperature; the reaction was stopped with 1 M sulfuric acid (Sigma-Aldrich). Optical density at 450 nm was measured using a microplate reader.

### 4.8. Western Blotting

Fifty micrograms of protein from cell lysates or skin samples were separated using sodium dodecyl sulfate polyacrylamide gel electrophoresis, transferred to a polyvinylidene fluoride membrane, and blocked in 5% skim milk to reduce nonspecific binding. After three washes, the membrane was incubated overnight at 4 °C with the appropriate dilution of primary antibody (Table S2). After further washes, the membrane was treated with a horseradish-peroxidase-conjugated secondary antibody (1:10,000; Vector Laboratories) for 1 h at room temperature. Protein bands were visualized using chemiluminescent solutions and captured with a ChemiDoc Imaging System.

### 4.9. Staining

#### 4.9.1. Oil Red O Staining

AD-MSCs were seeded at a density of 1 × 10^5^ cells per well in a 6-well plate. Cells were induced to differentiate by treatment with a 1:1 mixture of differentiation medium and CM. After 12 days, the cells were washed three times with PBS and fixed with 10% formalin at room temperature for 30 min. After fixation, cells were washed three times with distilled water, treated with propylene glycol (Abcam, Cambridge, UK) for 5 min, and then stained with Oil Red O solution (Abcam) for 1 h at room temperature. They were then treated with 85% propylene glycol for 30 s and washed twice with distilled water. Finally, the cells were counterstained with hematoxylin (Abcam) for 10 s, rinsed with tap water, mounted, and examined under a microscope.

#### 4.9.2. Immunohistochemistry

Paraffin-embedded sections were deparaffinized, antigen-retrieved in citrate buffer, and blocked. Slides were then incubated with the primary antibody overnight at 4 °C (Table S2). After additional PBS washes, slides were incubated with a biotinylated secondary antibody (Vector Laboratories) for 1 h at room temperature, followed by further PBS rinsing. Slides were then treated with an ABC reagent (Vector Laboratories) and washed. The 3,3′-diaminobenzidine (Sigma-Aldrich) substrate revealed positive staining as brown, and sections were counterstained with hematoxylin (KPNT, Cheongju, Republic of Korea). The stained tissue was scanned using a slide scanner (Motic Scan Infinity 100; Motic, Beijing, China), and images were captured.

#### 4.9.3. Hematoxylin and Eosin Staining

Deparaffinized slides were stained with hematoxylin (KPNT), rinsed, and then counterstained with eosin (KPNT). The slides were then dehydrated, mounted, and examined under a microscope. The stained tissue was scanned using a slide scanner (Motic Scan Infinity 100; Motic), and ten images were randomly captured.

### 4.10. Quantitative and Statistical Analysis

Quantification was conducted using ImageJ software version 1.53s (NIH); each group was compared with the control sample [79,80].

Data from three independent experiments were expressed as mean ± SD. Group differences were assessed using the Kruskal–Wallis test and Mann–Whitney U test for post hoc analysis, with statistical significance indicated in each figure legend. Analyses were performed using SPSS version 26 (IBM, Armonk, NY, USA).

## 5. Conclusions

This study elucidated the pivotal role of PDLLA in modulating adipogenesis to increase skin volume. Our findings highlight the intricate molecular mechanisms underlying the PDLLA-mediated rejuvenation of senescent macrophages. Specifically, we demonstrated that PDLLA initiates a signaling cascade through Piezo1 activation and M2 polarization, subsequently upregulating critical pathways, including FGF2, ERK1/2, PPAR-γ, and C/EBP-α. These signaling events lead to enhanced ASC proliferation and adipogenesis, suggesting a robust rejuvenation effect through increased adipose tissue thickness.

## Data Availability

All data are contained within this article.

## Supplementary Materials

The following supporting information can be downloaded at: https://www.mdpi.com/article/10.3390/ijms252312739/s1.
