# Supplementary material for: Poly-D,L-Lactic Acid Fillers Increase Subcutaneous Adipose Tissue Volume by Promoting Adipogenesis in Aged Animal Skin
Source: Int J Mol Sci. 2024 Nov 27;25(23):12739. doi: 10.3390/ijms252312739 (PMC11641794; doi:10.3390/ijms252312739)
Supplement: Supplementary file 1 [file ijms-25-12739-s001.zip › ijms-3335508-supplementary.pdf]

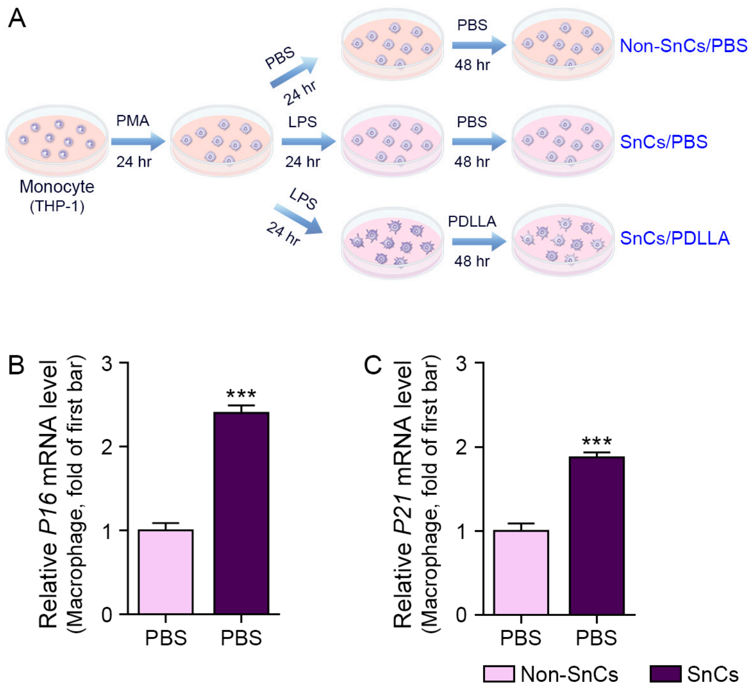

Figure S1. Regulation of senescence markers in senescent macrophages. (**A**) Schematic diagram of PDLLA treatment in senescent macrophages. (**B**,**C**) *P16* and *P21* mRNA level in senescent macrophages. Data are presented as the mean ± standard deviation of three independent experiments. ***, $p < 0.001$ vs. first bar. LPS, lipopolysaccharide; PDLLA, poly-D,L-lactic acid; PMA, 4β-phorbol-12-myristate-13-acetate, Non-SnCs; non-senescent macrophages SnCs; senescent macrophages.

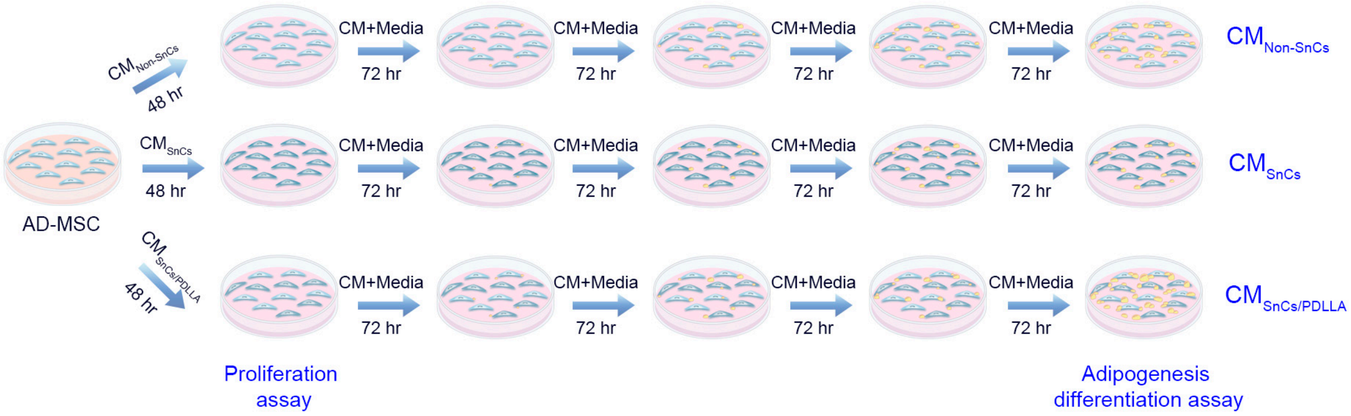

Figure S2. Schematic diagram of ASC treated with conditioned media for macrophages. CM, conditioned media. $CM_{Non\text{-}Sncs}$, CM from PBS-treated non-senescent macrophages; CM_{Sncs}, CM from PBS-treated senescent macrophages; $CM_{Sncs/PDLLA}$, CM from PDLLA-treated senescent macrophages; PDLLA, poly-D,L-lactic acid.

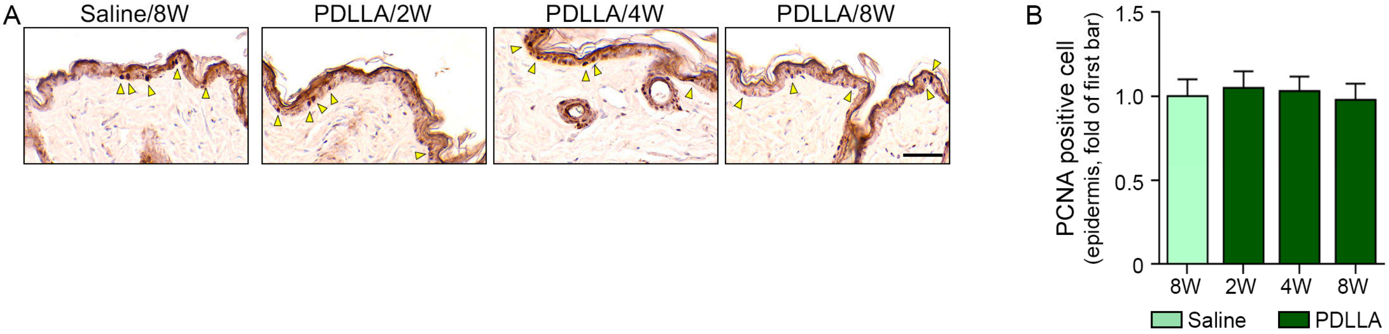

Figure S3. PCNA expression in epidermis of aged mice treated with PDLLA. Yellow mark is a positive signal. Data are presented as the mean ± standard deviation of three independent experiments. PCNA, proliferating cell nuclear antigen; PDLLA, poly-D,L-lactic acid.

Table S1. List of primers for quantitative reverse–transcription polymerase chain reaction.

Gene (Organism)	Primer sequences	
ACTB (human)	Forward	5'-CTC GCC TTT GCC GAT CC-3′
	Reverse	5'-TCT CCA TGT CGT CCC AGT TG-3′
P21 (human)	Forward	5'-GGA GAC TCT CAG GGT CGA AAA-3′
	Reverse	5'-GCT TCC TCT TGG AGA AGA TCA G-3′
P16 (human)	Forward	5'-CAC TTT CCT GGG CAA CAA ATA-3′
	Reverse	5'-CTT GCG GTC ATC ATC GTA GTT-3′

Table S2. List of antibodies for enzyme-linked immunosorbent assay (ELISA), western blot (WB) and immunocytochemistry (ICC) / immunohistochemistry (IHC).

Antibody	Company	Catalog No.	Dilution rate		
			ELISA	WB	ICC/IHC
FGF2	abcam	ab106245	1:500	-	-
Piezo1	Protein tech	15939-1-AP	-	1:500	-
CD80	Abclonal	A16039	-	1:1,000	
CD163	Santa cruz	Sc-58965	-	1:500	
ERK	cell signaling	9102s	-	1:1,000	-
pERK	Cell signaling	9101s	-	1:1,000	-
PCNA	GeneTex	GTX100539	-	1:500	1:100
PPAR-γ	Santa cruz	sc-7273	-	1:500	-
C/EBP-α	Biorbyt	orb672432	-	1:500	-
β-actin	Cell signaling	4967	-	1:1,000	-
